# Honokiol Alleviates Hypertrophic Scar by Targeting Transforming Growth Factor-β/Smad2/3 Signaling Pathway

**DOI:** 10.3389/fphar.2017.00206

**Published:** 2017-04-19

**Authors:** Danyang Zhao, Yu Wang, Chao Du, Shengzhou Shan, Yifan Zhang, Zijing Du, Dong Han

**Affiliations:** ^1^Department of Plastic and Reconstructive Surgery, Shanghai Ninth People’s Hospital, Shanghai Jiao Tong University School of MedicineShanghai, China; ^2^Department of Geriatrics, Shanghai Ninth People’s Hospital, Shanghai Jiao Tong University School of MedicineShanghai, China

**Keywords:** honokiol, hypertrophic scar, extracellular matrix, fibroblasts, TGF-β/Smad2/3

## Abstract

Hypertrophic scar (HPS) presents as excessive extracellular matrix deposition and abnormal function of fibroblasts. However, there is no single satisfactory method to prevent HPS formation so far. Here, we found that honokiol (HKL), a natural compound isolated from Magnolia tree, had an inhibitory effect on HPS both *in vitro* and *in vivo*. Firstly, HKL could dose-dependently down-regulate the mRNA and protein levels of type I collagen, type III collagen, and α-smooth muscle actin (α-SMA) in hypertrophic scar-derived fibroblasts (HSFs). Secondly, HKL suppressed the proliferation, migration abilities of HSFs and inhibited HSFs activation to myofibroblasts, but had no effect on cell apoptosis. Besides, the *in vivo* rabbit ear scar model further affirmed the inhibitory effects of HKL on collagen deposition, proliferating cell nuclear antigen and α-SMA. Finally, Western blot results showed that HKL reduced the phosphorylation status of Smad2/3, as well as affected the protein levels of matrix metalloproteinases (MMPs) and tissue inhibitor of metalloproteinase1. Taken together, this study demonstrated that HKL alleviated HPS by suppressing fibrosis-related molecules and inhibiting HSFs proliferation, migration as well as activation to myofibroblasts via Smad-dependent pathway. Therefore, HKL could be used as a potential agent for treating HPS and other fibrotic diseases.

## Introduction

Scarring is one of the normal complications of tissue repair (Bayat et al., 2003). Traumas that deep into dermis can easily lead to hypertrophic scar (HPS) formation. The clinical manifestations of HPS are prominent surface, irregular shape, rugged, flush with blood, real toughness, accompanied by burning pain and itch, all these seriously affect the life qualify of patients. As a type of excessive fibrotic disease, HPS not only damages the shape of the contour, but also causes functional impairment when occurs at the joint place (Rockwell et al., 1989; Rabello et al., 2014; Zielins et al., 2014). Nowadays, there are several therapeutic interventions for HPS, including surgery, corticosteroid combined with 5-fluorouracil injection, pressure and laser radiation therapy, but the treatment effects are not satisfactory (Zurada et al., 2006; Arno et al., 2014).

Inflammation, tension, neural factors, and cytokines are considered to be associated with the formation of HPS, however, the pathogenesis of HPS has not been fully elucidated. It is found that scar hyperplasia is a common result of overproduction of various cytokines, abnormal proliferation, activation, contraction of fibroblasts, and collagen deposition (Wolfram et al., 2009). Transforming growth factor-beta (TGF-β) plays a critical role on the pathological process of HPS, it mediates fibroblasts proliferation, myofibroblasts differentiation, and extracellular matrix (ECM) deposition, and targeting TGF-β through different pathways can prevent HPS formation (Beanes et al., 2003; Chalmers, 2011; Lian and Li, 2016).

Honokiol (HKL), or 3′,5-di-(2-propenyl)-1, 1′-biphenyl-2, 4′-diol, is a kind of small molecule polyphenol isolated from the bark of Magnolia tree. Modern pharmacological researches have suggested that HKL has anti-inflammatory, anti-bacterial, anti-oxidant, anti-tumor, anti-calmodulin, neuroprotective, and other effects (Fried and Arbiser, 2009; Shen et al., 2010; Lee et al., 2011; Woodbury et al., 2013; Pan et al., 2016). A recent study had reported that HKL could reverse cardiac hypertrophy by inhibiting cardiac fibroblasts proliferation and differentiation to myofibroblasts (Pillai et al., 2015). Besides, HKL was believed to have the abilities to suppress the expression of pro-fibrotic factors and ECM proteins, which could be used as a therapeutic target for the treatment of renal fibrosis (Chiang et al., 2011). Based on these efficacies, we made a hypothesis that HKL could also act on hypertrophic scar-derived fibroblasts (HSFs) so as to alleviate scar hyperplasia.

## Materials and Methods

### Cell Culture

Hypertrophic scar samples were collected from patients in Department of Plastic and Reconstructive Surgery, Shanghai Ninth People’s Hospital, Shanghai Jiao Tong University School of Medicine with written consents in accordance with the Helsinki Declaration. None of the patients had received treatments before surgery. After trimming the subcutaneous adipose tissue, scar samples were immersed in 0.25% dispase II (Roche, Germany) at 4°C overnight. Then, the dermis was separated from epidermis and digested with 0.25% collagenase I (Sigma, USA) at 37°C for 4–6 h. Isolated HSFs were cultured in complete high glucose Dulbecco modified eagle medium (DMEM, Hyclone, USA) supplemented with 10% fetal bovine serum (FBS, Gibco, USA), 100 U/ml penicillin and 100 μg/ml streptomycin (Hyclone, USA). Cells from third to sixth passages were obtained for all the experiments.

### Treatment of HSFs

Cells that reached 80–90% confluence were digested with 0.25% trypsin (Invitrogen, USA) and seeded in 6-well plates (1 × 10^5^/ml, 2 ml/well). Fifteen wells were divided into five groups (*n* = 3) with each being treated with HKL at 0, 2, 4, 6 or 8 μg/ml. Eighteen wells were divided into six groups (*n* = 3) with each being treated with HKL (0 μg/ml), TGF-β1 (5 ng/ml), TGF-β1 (5 ng/ml) + HKL (2 μg/ml), TGF-β1 (5 ng/ml) + HKL (4 μg/ml), TGF-β1 (5 ng/ml) + HKL (6 μg/ml) or TGF-β1 (5 ng/ml) + HKL (8 μg/ml). HKL was purchased from Selleck Chemicals Corporation (USA) and dissolved in dimethyl sulfoxide (DMSO) to a final stock concentration of 40 mg/ml. Human TGF-β1 (Peprotech, USA) was prepared at the concentration of 20 ng/μl.

### Quantitative Real-time PCR (qRT-PCR)

Total RNA was extracted from HSFs with different treatments for 48 h by TRI reagent (Molecular Research Center, USA). Spectrophotometer (NanoDrop2000, USA) was then applied to detect the purity of RNA. Synthesize of cDNA with 1.0 μg RNA. The primer sequences used in this study were described as follows: collagen, type I, alpha 1 (COL1A1), 5′-GTGCGATGACGTGATCTGTGA-3′ (forward), and 5′-CGGTGGTTTCTTGGTCGGT-3′ (reverse); collagen, type I, alpha 2 (COL1A2), 5′-GAGCGGTAACAAGGGTGAGC-3′ (forward), and 5′-CTTCCCCATTAGGGCCTCTC-3′ (reverse); collagen, type III, alpha 1 (COL3A1), 5′-TTGAAGGAGGATGTTCCCATCT-3′ (forward), and 5′-ACAGACACATATTTGGCATGGTT-3′(reverse); alpha-smooth muscle actin (α-SMA), 5′-GTGTTGCCCCTGAAGAGCAT-3′ (forward), and 5′-GCTGGGACATTGAAAGTCTCA-3′ (reverse); glyceraldehyde phosphate dehydrogenase (GAPDH), 5′-ACAACTTTGGTATCGTGGAAGG-3′ (forward), and 5′-GCCATCACGCCACAGTTTC-3′ (reverse). Gene expression level of COL1A1, COL1A2, COL3A1, and α-SMA were amplified by qRT-PCR using SYBR^®^ Premix Ex Taq^TM^ Kit (Takara, Japan) and normalized to GAPDH.

### Western Blot

Three days after different treatments, HSFs were lysed in radioimmunoprecipitation assay (RIPA) lysis buffer with 1 mM phenylmethyl sulfonyl fluoride (PMSF) for 30 min on ice and centrifuged at 1,2000 rpm, 4°C for 10 min. Collected supernatant and then detected the concentration of protein by BCA Protein Assay Kit (Thermo Fisher Scientific, USA). Thirty microgram of protein extract was separated by 10% sodium dodecyl sulfate-polyacrylamide gel electrophoresis (SDS-PAGE) and transferred to polyvinylidene difluoride (PVDF) membrane (Millipore, USA). After blocking with 5% bovine serum albumin (BSA), the membrane was immunoblotted with primary antibodies at 4°C overnight. The primary antibodies were anti-type I collagen (COL I, Genetex, USA), anti-matrix metalloproteinases (MMPs), anti-tissue inhibitor of metalloproteinase1 (TIMP1), anti-type III collagen (COL III), anti-α-SMA (Abcam, UK), anti-TGF-β1, anti-TGF-β receptor I (TGFβRI) and anti-TGF-β receptor II (TGFβRII), anti-phospho-Smad2/3 (anti-p-Smad2/3), anti-Smad2/3 (Cell signaling technology, USA) antibodies. Horseradish peroxidase (HRP)-conjugated secondary antibodies (Cell Signaling Technology, USA) were incubated with membrane at room temperature for 1 h on the second day. The protein expression levels were detected by Immobilon Western Chemiluminescent HRP Substrate (Millipore, USA) and analyzed with the Gelpro software. Reference gene of GAPDH (Abcam, UK) was used as loading control.

### Cell Counting Kit-8 (CCK-8) Assay

The proliferation of HSFs was measured by CCK-8 assay. Briefly, HSFs were seeded in 96-well plates (1 × 10^5^/ml, 100 μl/well) and treated with HKL at 0, 2, 4, 6, or 8 μg/ml. Five replicates were made for each concentration, and the medium was changed every 2 days. After treatment for 1, 2, 3, 4, and 5 days, 10 μl CCK-8 solution (Dojindo, Japan) was added into each well, followed by incubation for 2 h in 37°C incubator, the cells viability were quantified by the absorbance at 450 nm.

### Wound Healing Assay

Wound healing assay reflects the migration behavior of HSFs. First of all, HSFs were seeded in 6-well plates with complete medium. When reached confluence, cells were dealt with serum-depleted medium for another 12 h. A scratch wound was then made in the middle of each well by 1 ml pipette tips. After washing three times with phosphate buffer saline (PBS), HSFs were treated with HKL at 0 μg/ml or 6 μg/ml for 1 day. The gap of scratch was recorded at 0, 6, 12, 18, and 24 by inverted phase microscope (Nikon, Japan). Finally, using Image-Pro Plus system to analyze the wound area.

### Transwell Assay

Migration ability of HSFs could also be assessed by transwell assay using Millicell Hanging Cell Culture Insert (Millipore, USA). Briefly, HSFs suspension (1 × 10^5^/ml, 200 μl without FBS) was added into the inserts and then 700 μl HKL at 0 μg/ml or 6 μg/ml were added into the bottom wells supplemented with 10% FBS. After 24 h of incubation, the inserts were taken out of the 24-well plates, fixed with 4% paraformaldehyde for 5 min, and stained with crystal violet for 10 min. Non-migrated cells on the upper layer were gently wiped out by cotton balls. The number of migrated cells per field was quantified under microscope.

### Annexin V/Propidium Iodide Staining

The effect of HKL on cell apoptosis was evaluated by the technology of flow cytometry (Beckman, USA). In brief, HSFs were seeded in 6-well plates and treated with HKL at 0, 2, 4, 6, or 8 μg/ml for 2 days. Afterward, HSFs were digested and washed with cold PBS for three times. Cell apoptosis was detected using Alexa Fluor^®^ 488 annexin V/Dead Cell Apoptosis Kit (Invitrogen, USA) according to the experimental instructions. The stained cells were then analyzed by FlowJo software.

### Immunofluorescence

Hypertrophic scar-derived fibroblasts in confocal culture dishes were fixed with 4% paraformaldehyde for 15 min, permeabilized with 0.5% Triton-X100 (Sigma, USA) for 20 min and blocked with 5% donkey serum (Jackson, USA) for 1 h. After that, cells were incubated with mouse monoclonal anti-α-SMA primary antibody (Abcam, UK) at 4°C overnight. On the following day, cells were incubated with Alexa Fluor^®^ 594 donkey anti-mouse IgG secondary antibody (Invitrogen, USA) for 1 h at room temperature. The nuclei were stained with Hoechst (Invitrogen, USA). Zeiss laser-scanning microscope was used to analyze the fluorescence.

### Animal Model

The animal experiments and surgical procedures were approved by Shanghai Jiao Tong University School of Medicine and conducted in accordance with the “Guide for Care of Laboratory Animals” outlined by the National Ministry of Science. Twelve male New Zealand white rabbits (6–8 weeks old, 2.5–3.0 kg) were maintained under standard conditions and received human care.

Animals were anesthetized preoperatively with an intravenous administration of 1.5% pentobarbital (2 ml/kg) into the lateral ear vein. Ears were then shaved and disinfected with 75% ethyl alcohol. Four full-thickness dermal defects were created on each ear of twelve rabbits by an 8-mm biopsy punch, each defect was down to the bare cartilage on the ventral side. The perichondrium should be completely removed and hemostasis should be paid attention to. Twenty-four hours after surgery, each wound on the left ear of 12 rabbits was injected with DMSO (0.02%) as control while the right ear was injected with HKL (8 μg/ml) every other day. Both the DMSO and HKL were dissolved in normal saline and injected at the peripheries of each wound with 100 μl.

Animals were sacrificed, respectively at day 14 (*n* = 6) and day 28 (*n* = 6). The full-thickness scars and the surrounding tissue were obtained together. A caliper was used to measure the maximum protuberant heights of HPS and the normal skin. HPS can be quantified by scar elevation index (SEI), which is the ratio of total height of wound area to that of normal tissue.

### Sirius Red Staining

The specimens were fixed with 4% paraformaldehyde for 24 h and rinsed under running water overnight. After dehydrated in graded ethanol, the paraffin-embedded tissue was cut into 5 μm-thick slides. Tissue sections were stained with Sirius red (Fluka, Switzerland) and observed by polarized light microscope (Zeiss, Germany). Collagen fibers were measured and analyzed by Image-Pro Plus system.

### Immunohistochemistry (IHC)

Immunohistochemistry (IHC) was performed to detect proliferating cell nuclear antigen (PCNA) and α-SMA. The deparaffinized sections were repaired with pepsin (Sigma, USA) at 37°C for 30 min, blocked endogenous peroxidase activity with 3% H_2_O_2_, and incubated with mouse monoclonal anti-PCNA antibody and anti-α-SMA antibody (Abcam, UK) at 4°C overnight. Negative control was treated with PBS. Then the sections were incubated with goat anti-mouse IgG-HRP (Maixin, China) at room temperature for 1 h. Finally, the staining was visualized with DAB Detection Kit (Maixin, China) and counterstained with hematoxylin.

### TUNEL Assay

Cell apoptosis *in vivo* was detected by terminal deoxynucleotidyl transferase-mediated dUTP nick end labeling (TUNEL) assay using the In Situ Cell Death Detection Kit (Roche, Germany). The percent of TUNEL positive cells were quantified by Image-Pro Plus system.

### Statistical Analysis

Results were presented as mean ± standard deviation (SD). The data were analyzed for significance by the method of Student’s *t*-test, and *P* < 0.05 was considered as statistically different.

## Results

### HKL Dose-dependently Reduced mRNA and Protein Levels of COL I, COL III, and α-SMA in HSFs

**FIGURE 1:**
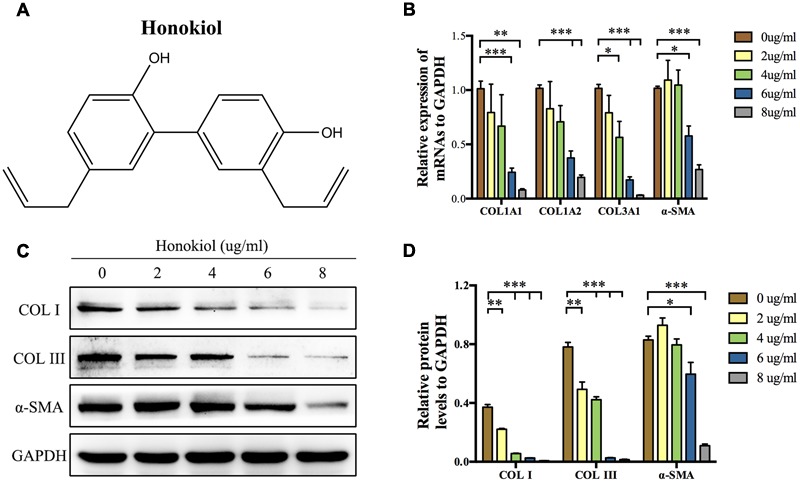
**Effects of HKL on mRNA and protein levels of fibrosis-related molecules. (A)** Chemical structure of HKL. **(B)** qRT-PCR results of the mRNA levels of COL1A1, COL1A2, COL3A1, and α-SMA after HKL treatment for 2 days at 0, 2, 4, 6, or 8 μg/ml. GAPDH served as control. *n* = 3. **(C)** Western blot results of protein levels of COL I, COL III, and α-SMA after HKL treatment for 3 days at 0, 2, 4, 6, or 8 μg/ml. GAPDH was used as loading control. **(D)** Quantification of protein levels in **(C)** which normalized to the level of GAPDH. *n* = 3. Each bar shows as mean ± SD. ^∗^*P* < 0.05; ^∗∗^*P* < 0.01; ^∗∗∗^*P* < 0.001.

COL I, COL III, and α-SMA are three of fibrosis-related molecules that highly expressed in HPS (Bhogal et al., 2005; Armour et al., 2007; Wang et al., 2011). In order to investigate the effect of HKL (chemical structure was shown in **Figure 1A**) on the expression of these fibrosis-related molecules in HPS, HFSs were cultured and treated with HKL at 0, 2, 4, 6, or 8 μg/ml. The mRNA and protein levels of these fibrosis-related molecules were assessed by qRT-PCR and Western blot. On the one hand, qRT-PCR results showed that HKL dose-dependently down-regulated the mRNA expression of COL1A1, COL1A2, COL3A1, and α-SMA. HKL could significantly reduce the mRNA levels of COL1A1, COL1A2, and α-SMA from 6 μg/ml, and reduce the COL3A1 mRNA level from 4 μg/ml (**Figure 1B**). On the other hand, the results of Western blot almost showed the same tendency. HKL remarkably suppressed the protein levels of COL I and COL III at the beginning concentration of 2 μg/ml, however, the protein expression of α-SMA was inhibited from 6 μg/ml (**Figures 1C,D**). Therefore, it is suggested that HKL could dose-dependently reduce COL I, COL III, and α-SMA expressions in HSFs, and the concentration of 6 μg/ml was regarded as the lowest effective concentration and used in the following experiments. In order to investigate whether the organic solvent affected the experimental results or not, we compared the highest concentration of 0.02% DMSO (HKL at 8 μg/ml) with HKL at 0 μg/ml to the expression of fibrosis-related molecules, and the results showed that solvent could not affect the expression of the above-mentioned molecules in HSFs (**Supplementary Figures S1A–C**). All these results demonstrated that HKL could inhibit the expression of fibrosis-related molecules in HSFs *in vitro*.

### HKL Affected the Proliferation and Migration Abilities of HSFs *In Vitro* But Had No Impact on Cell Apoptosis

**FIGURE 2:**
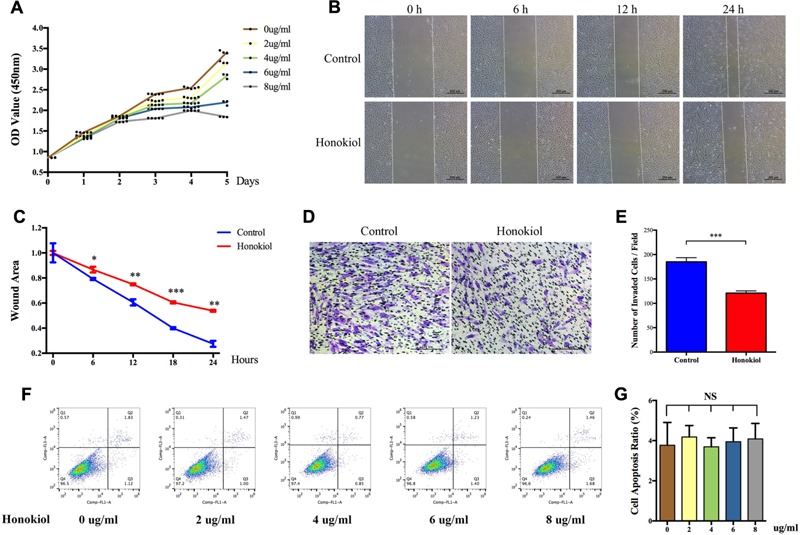
**Effects of HKL on HSFs proliferation, migration, and apoptosis. (A)** Proliferation curves of HSFs after treating with HKL at 0, 2, 4, 6, or 8 μg/ml for 1, 2, 3, 4, and 5 days by CCK-8 assay. **(B)** Representative pictures of the wound-healing assay after HKL treatment at 0 μg/ml or 6 μg/ml for 0, 6, 12, and 24 h. The white dotted lines indicate wound scratch. **(C)** The wound area quantified of **(B)**. *n* = 3. **(D)** Representative pictures of the transwell assay after HKL treatment at 0 μg/ml or 6 μg/ml for 24 h. **(E)** The number of invaded HSFs per field quantified of **(D)**. *n* = 3. **(F)** Flow cytometry results of cell apoptosis after treatment of HKL for 2 days at 0, 2, 4, 6, or 8 μg/ml. **(G)** Quantification of cell apoptosis ratio in **(F)**. *n* = 3. Data are mean ± SD. ^∗^*P* < 0.05; ^∗∗^*P* < 0.01; ^∗∗∗^*P* < 0.001. OD value, optical density value; NS, no significance.

Proliferation and migration abilities of HSFs are closely associated with scar hyperplasia, and inhibiting the proliferation and migration of fibroblasts has therapeutic effect on HPS (Bai et al., 2015; Wang et al., 2015; Li et al., 2016). Therefore, we investigated the effect of HKL on the proliferation and migration of fibroblasts. CCK-8 assay was performed to analysis the influence of HKL on HSFs proliferation. As shown by proliferation curves, HKL dose-dependently inhibited the proliferation ability of HSFs from 2 μg/ml to 8 μg/ml (**Figure 2A**). Next, we conducted wound healing assay and transwell assay to examine whether HKL (6 μg/ml) could influence HSFs migration ability. **Figure 2B** showed that wound clearance of the HKL treated group was wider at 6, 12, and 24 h compared with that of the control group, and the wound area also statistically increased under the treatment of HKL (**Figure 2C**). Crystal violet staining indicated that there were less migrated HSFs after the treatment of HKL (**Figure 2D**), and the number of invaded cells in the HKL treated group were significantly decreased compared with that of control group (**Figure 2E**). In addition, flow cytometry analysis manifested that different concentrations of HKL failed to affect cell apoptosis of HSFs (**Figures 2F,G**). Furthermore, the highest concentration of 0.02% DMSO did not affect the proliferation ability and cell apoptosis of HSFs (**Supplementary Figures S1D–F**). These results revealed that HKL could prohibit the proliferation and migration of HSFs but could not influence the cells apoptosis *in vitro*.

### HKL Suppressed HSFs Activation

**FIGURE 3:**
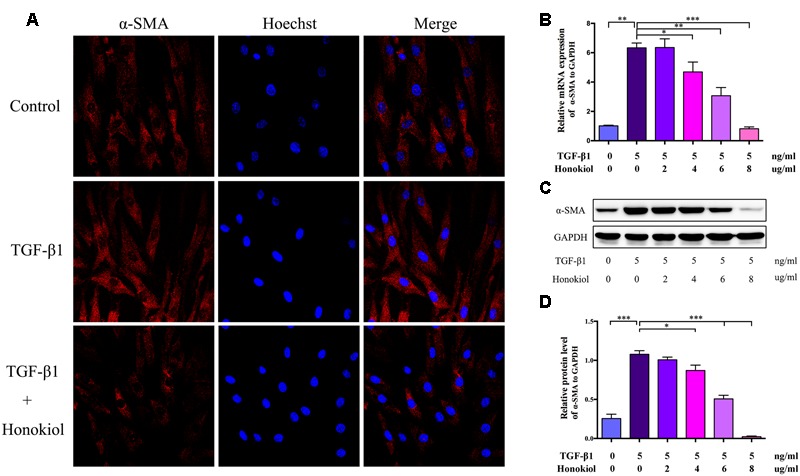
**HKL suppressed HSFs activation *in vitro*. (A)** Immunofluorescence staining for α-SMA in HSFs after treating with HKL (0 μg/ml), TGF-β1 (5 ng/ml), TGF-β1 (5 ng/ml) + HKL (6 μg/ml) for 2 days. α-SMA is shown by red fluorescence. Nuclei were stained with Hoechst and shown by blue fluorescence. **(B)** qRT-PCR results of α-SMA mRNA level after treatment of HKL (0 μg/ml), TGF-β1 (5 ng/ml), TGF-β1 (5 ng/ml) + HKL (2 μg/ml), TGF-β1 (5 ng/ml) + HKL (4 μg/ml), TGF-β1 (5 ng/ml) + HKL (6 μg/ml) or TGF-β1 (5 ng/ml) + HKL (8 μg/ml) for 2 days. GAPDH served as control. *n* = 3. **(C)** Western blot results α-SMA protein level after treatment of HKL (0 μg/ml), TGF-β1 (5 ng/ml), TGF-β1 (5 ng/ml) + HKL (2 μg/ml), TGF-β1 (5 ng/ml) + HKL (4 μg/ml), TGF-β1 (5 ng/ml) + HKL (6 μg/ml) or TGF-β1 (5 ng/ml) + HKL (8 μg/ml) for 3 days. GAPDH was used as loading control. **(D)** Quantification of α-SMA protein level in **(C)** which normalized to the level of GAPDH. *n* = 3. Each bar shows as mean ± SD. ^∗^*P* < 0.05; ^∗∗^*P* < 0.01; ^∗∗∗^*P* < 0.001.

Fibroblasts can be activated by TGF-β1 to differentiate into myofibroblasts. Myofibroblasts, as the activated fibroblasts, contribute to scar contraction and marked by high expression of α-SMA (Ehrlich et al., 1994; Moreels et al., 2008; Wang et al., 2011; Chrysanthopoulou et al., 2014). For the purpose of discussing the role of HKL on TGF-β1-induced activation, HSFs were treated with HKL (0 μg/ml), TGF-β1 (5 ng/ml), TGF-β1 (5 ng/ml) + HKL (2 μg/ml), TGF-β1 (5 ng/ml) + HKL (4 μg/ml), TGF-β1 (5 ng/ml) + HKL (6 μg/ml) or TGF-β1 (5 ng/ml) + HKL (8 μg/ml). The immunocytofluorescence staining showed that HKL (6 μg/ml) remarkably down-regulated TGF-β1-induced up-regulation of α-SMA (**Figure 3A**). We further verified the effect of HKL on HSFs activation by qRT-PCR and Western blot, and results showed that the raised mRNA and protein levels of α-SMA induced by 5 ng/ml TGF-β1 were significantly suppressed by HKL from the concentration of 4 μg/ml (**Figures 3B–D**). These results indicated that HKL could effectively suppress the activation of HSFs.

### HKL Alleviated HPS *In Vivo*

**FIGURE 4:**
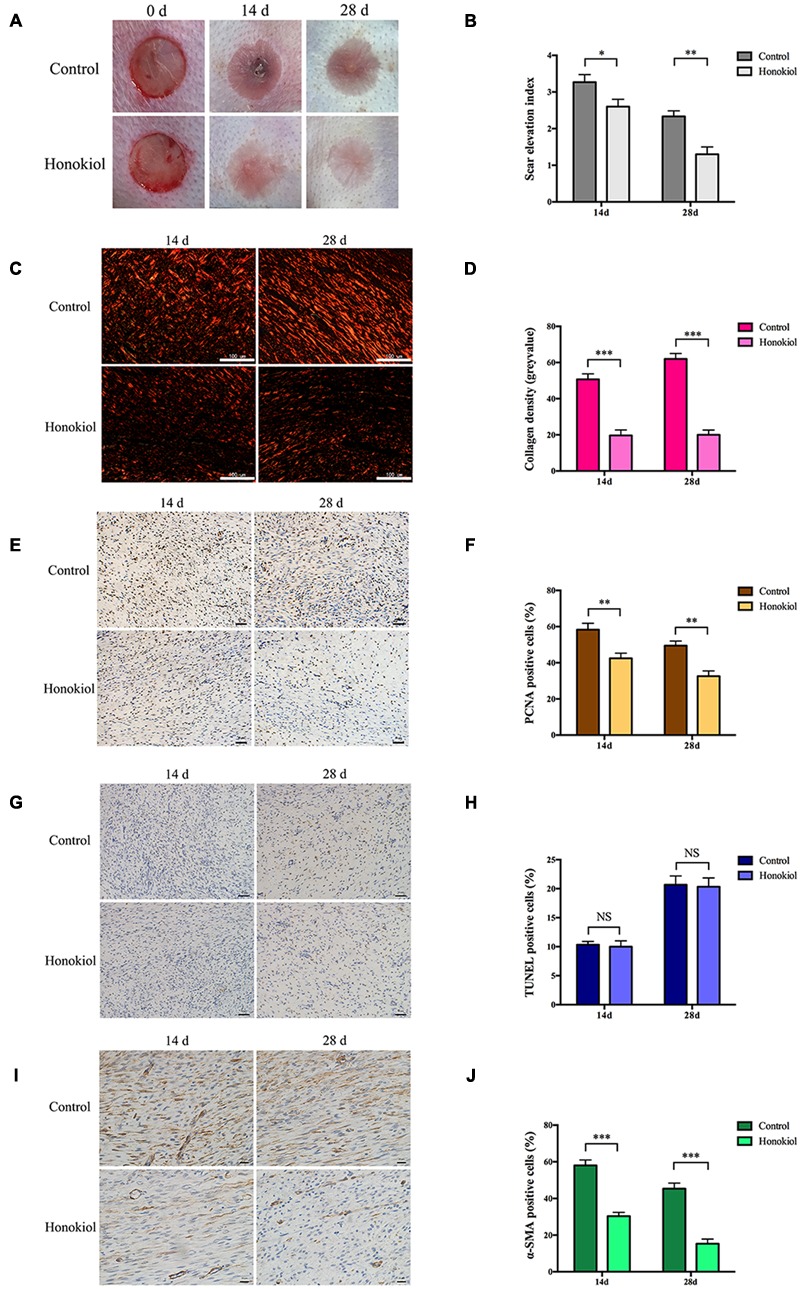
**HKL alleviated HPS formation in rabbit ear scar model. (A)** Representative photos of rabbit ear scars at day 14 and day 28 in the control group and the HKL group. **(B)** SEI quantification of DMSO-treated scars and HKL-treated scars of 14 and 28 days after operation. *n* = 6. **(C)** Sirius red staining of HPS under polarized light. **(D)** Quantification of collagen density measured in **(C)**. *n* = 6. **(E)** IHC images of HPS highlighting PCNA positive cells after treatment of DMSO or HKL at day 14 and day 28. **(F)** Quantification of the percentage of PCNA positive cells showed in **(E)**. *n* = 6. **(G)** Images of TUNEL assay after treatment of DMSO or HKL at day 14 and day 28. **(H)** Quantification of the percentage of TUNEL positive cells showed in **(G)**. *n* = 6. **(I)** Representative images of IHC staining indicating α-SMA positive cells of DMSO group and HKL group at day 14 and day 28. **(J)** Quantification of the percentage of α-SMA positive cells showed in **(I)**. *n* = 6. Data are mean ± SD. ^∗^*P* < 0.05; ^∗∗^*P* < 0.01; ^∗∗∗^*P* < 0.001.

Our *in vitro* data demonstrated that HKL could inhibit the expression of collagen and suppress the proliferation, migration, and activation abilities of HSFs. We next investigated the effect of HKL on HPS *in vivo* in a rabbit ear scar model. First of all, we recorded images of scars and SEI at day 14 and day 28. Results showed that HKL (8 μg/ml) treated ears presented an alleviated scar formation compared with DMSO treated ears (**Figure 4A**). Moreover, SEI was significantly decreased in HKL treated group than that of DMSO treated group (**Figure 4B**), which demonstrated that HKL could inhibit HPS formation.

Next, Sirius red staining was conducted to detect the content of collagen, and the collagen density was significantly lower in HKL treated group than that of control group both on 14 and 28 days (**Figures 4C,D**). In addition, the proliferation behavior of HSFs was also assessed by IHC *in vivo*, HKL-treated scar sections exhibited significantly decreased PCNA positive cells compared with that of DMSO-treated scar both on day 14 and day 28 (**Figures 4E,F**). However, there was no significant difference for TUNEL positive cells between scars treated with HKL or DMSO (**Figures 4G,H**), which indicated that HKL failed to affect cell apoptosis *in vivo*. Finally, both the staining and quantification results of IHC showed that α-SMA positive cells of the HKL-injected scars were notably decreased compared with the DMSO-injected scars (**Figures 4I,J**). These results were consistent with our studies *in vitro*.

### HKL Down-regulated the Protein Level of p-Smad2/3 As Well As Affected the Expression of MMPs and TIMP1 in HSFs

As proved above, HKL could inhibit collagen deposition as well as the proliferation, migration, and activation abilities of HSFs both *in vitro* and *in vivo*. We further explored the underling mechanism using Western blot.

**FIGURE 5:**
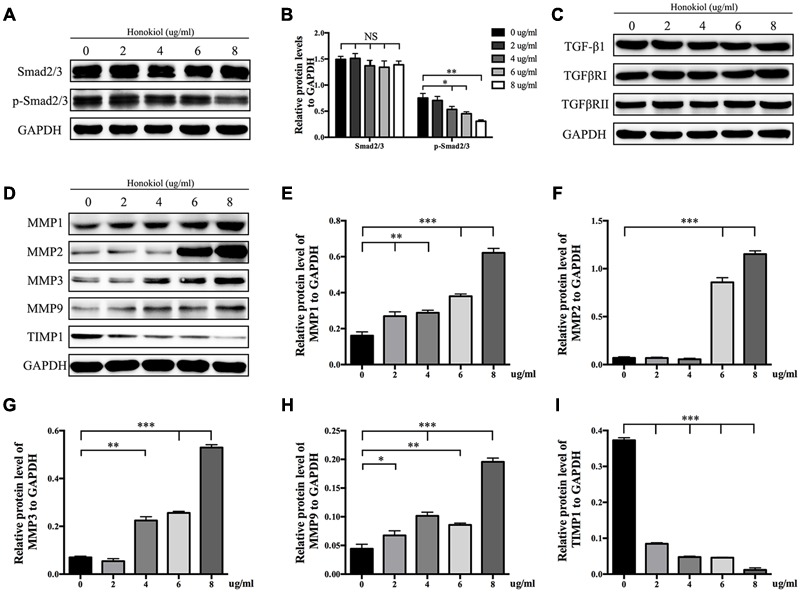
**HKL inhibited phosphorylation of Smad2/3 and affected protein levels of MMPs and TIMP1. (A)** Protein levels of total Smad2/3 and p-Smad2/3 of HSFs after HKL treatment at 0, 2, 4, 6, or 8 μg/ml for 3 days. GAPDH served as control. **(B)** Quantification of protein levels of total Smad2/3 and p-Smad2/3 which normalized to the level of GAPDH. *n* = 3. **(C)** Results of western blot showing levels of TGF-β1, TGFβRI, and TGFβRII with different HKL treatments. GAPDH served as control. **(D)** Western blot results showing the protein level changes of MMP1, MMP2, MMP3, MMP9, and TIMP1 after HKL treatments in HSFs. GAPDH served as control. **(E)** Quantification of protein level of MMP1 showed in **(D)**. *n* = 3. **(F)** Quantification of protein level of MMP2 showed in **(D)**. *n* = 3. **(G)** Quantification of protein level of MMP3 showed in **(D)**. *n* = 3. **(H)** Quantification of protein level of MMP9 showed in **(D)**. *n* = 3. **(I)** Quantification of protein level of TIMP1 showed in **(D)**. *n* = 3. Each bar shows as mean ± SD. ^∗^*P* < 0.05; ^∗∗^*P* < 0.01; ^∗∗∗^*P* < 0.001.

It is widely known that TGF-β takes part in various fibrotic diseases, including HPS (Border and Noble, 1994; Branton and Kopp, 1999; Ihn, 2002; Pohlers et al., 2009). We have already confirmed that HKL suppressed the elevated expression of α-SMA induced by TGF-β1 (**Figure 3**). On the basis of the results mentioned above, the downstream agents of TGF-β signaling pathway were selectively investigated. As shown in **Figures 5A,B**, the phosphorylation status of Smad2/3 was dose-dependently down-regulated by HKL from 0 to 8 μg/ml while the total Smad2/3 level did not change under the same treatments. However, HKL showed no obvious effect on the level of TGF-β1 secretion (**Figure 5C**). Next, we detected the levels of TGFβRI and TGFβRII, which help to phosphorylate Smad2/3 (Kamato et al., 2013), however, it had the same results as TGF-β1 (**Figure 5C**). These results demonstrated that HKL could suppress the phosphorylation of Smad2/3, but the inhibitory effect of HKL on p-Smad2/3 level was independent of TGF-β1, TGFβRI, and TGFβRII levels.

Finally, we analyzed the expression of MMPs and TIMP1, which associated with the degradation of ECM. The Western blot and quantification results showed that HKL significantly up-regulated the protein levels of MMP1 and MMP9 while down-regulated the protein level of TIMP1 at the beginning concentration of 2 μg/ml, moreover, HKL remarkably increased the protein levels of MMP2 and MMP3, respectively, from 6 and 4 μg/ml (**Figures 5D–I**).

## Discussion

Hypertrophic scar is an inevitable fibrotic consequence caused by trauma, surgery, burn, and inflammation. It presents as an abnormal healing process that characterized by excessive fibrosis and aberrant ECM deposition (Armour et al., 2007; Arno et al., 2014). However, the therapeutic strategies for scarring were limited and barely had satisfactory outcomes (Zurada et al., 2006).

Fibroblasts, as the main component cells of dermis, take part in the process of wound healing and tissue remolding after skin injuries. During the pathological process of scar formation, fibroblasts were activated to differentiate into myofibroblasts which express excessive α-SMA, and produce massive ECM such as COL I and COL III (Armour et al., 2007; Chrysanthopoulou et al., 2014). In this study, we simultaneously applied HSFs and rabbit ear scar model to investigate the anti-scar effect of HKL. The results showed that HKL could alleviate HPS not only by inhibiting the collagen deposition, but also by suppressing the proliferation, migration and activation abilities of HSFs both *in vitro* and *in vivo*, which revealed the potential of HKL for scar treatment. This finding is also in line with previous publications demonstrating the anti-fibrosis effect of HKL on other diseases, including cardiac hypertrophy and renal fibrosis (Chiang et al., 2011; Pillai et al., 2015).

Transforming growth factor-beta, as a multifunctional cytokine which takes part in regulating cells growth, differentiation and immunity, plays an important role on various tissue fibrotic diseases, such as kidney fibrosis, lung fibrosis, hepatic fibrosis, and cardiac fibrosis (Border and Noble, 1994; Lijnen et al., 2000; Liu et al., 2006; Choi et al., 2012; Tatler and Jenkins, 2012). It is also related to skin fibrosis as it induces the ECM deposition and fibroblasts activation (Chalmers, 2011). Studies have reported that fibrosis could be inhibited by targeting TGF-β/Smad2/3 signaling pathway (Xiao et al., 2014; Bai et al., 2015; Zhang Y. et al., 2016; Zhang Y.F. et al., 2016). The Western blot results showed that HKL dose-dependently suppressed the p-Smad2/3 level, while the total TGF-β1 and Smad2/3 levels were unaffected, these indicated that the inhibitory effect of HKL was via Smad-dependent pathway (**Figures 5A,B**). It is reported that TGF-βRI can work with TGF-βRII to phosphorylate Smad2/3 (Kamato et al., 2013), thus we were to investigate the expression of TGFβRI and TGFβRII. Interestingly, there was no significant change of TGFβRI and TGFβRII expression after treated with HKL, then we drew the conclusion that the inhibitory effect of HKL on p-Smad2/3 level was irrelevant to TGF-β1, TGFβRI, and TGFβRII levels (**Figure 5C**). Our results suggested that HKL could alleviate scar hyperplasia through inhibiting phosphorylation of Smad2/3, but the underlying mechanism might not lead to any change in TGF-β1, TGFβRI, and TGFβRII expression. The study of special transcription factors which involved in regulating the phosphorylation of smad2/3 will be undertaken in our future research.

Matrix metalloproteinases play key roles on scar remolding, which are not only involved in ECM degradation, but also related to cells migration during wound repair (Page-McCaw et al., 2007; Giannandrea and Parks, 2014; Rohani and Parks, 2015). TIMPs, which reverse the effect of MMPs, work together with MMPs to maintain the balance of collagen in the wound environment (Hemmann et al., 2007; Arpino et al., 2015). Some publications demonstrated that HKL could inhibit the expression of MMPs in other diseases, such as arthritis and cancer (Ahn et al., 2006; Kim et al., 2010; Chen et al., 2014; Zhang et al., 2015), however, the results of our study displayed that HKL could up-regulate the protein expression of MMP1, MMP2, MMP3, and MMP9 while down-regulate the protein expression of TIMP1 both at the lowest effective concentration at 6 μg/ml (**Figures 5D–I**). It suggested that HKL inhibits HPS formation by regulating the degradation of ECM mediated by MMPs and TIMP1, although the underlying mechanism is not clear.

To sum up, we are the first to demonstrate that HKL could prevent scarring by inhibiting the proliferation, migration and activation abilities of HSFs both *in vitro* and *in vivo.* In addition, this work further demonstrated that HKL alleviated HPS formation by inhibiting TGF-β/Smad2/3 signaling pathway through a dependent way. Taken together, it suggested that HKL could be used as a potential anti-fibrosis drug for HPS and other fibrotic diseases.

## Author Contributions

DZ and YW conceived and designed the experiment. DZ, YW, and CD performed the experiment. SS, YZ, and ZD helped for the experiment and gave advises. DZ wrote the paper, YW and CD then took part in analyzing the revision of the manuscript. DH has been the corresponding author and provided technical assistance throughout the experiment and writing process. All authors reviewed the results and approved the final version of the manuscript.

## Conflict of Interest Statement

The authors declare that the research was conducted in the absence of any commercial or financial relationships that could be construed as a potential conflict of interest.

## Supplementary Material

FIGURE S1 **Effects of the solvent DMSO at its highest concentration of 0.02% used for HKL preparation. (A)** qRT-PCR results of the mRNA levels of COL1A1, COL1A2, COL3A1, and α-SMA after treatment of 0 μg/ml HKL and 0.02% DMSO. GAPDH served as control. *n* = 3. **(B)** The effect of 0.02% DMSO on protein levels of COL I, COL III and α-SMA in HSFs by Western blot. GAPDH served as control. **(C)** Quantification of protein levels in **(B)** which normalized to the level of GAPDH. *n* = 3. **(D)** Assessment of the effect of 0.02% DMSO on HSFs proliferative activity by CCK-8 assay. **(E)** Assessment of the effect of 0.02% DMSO on cell apoptosis by flow cytometry. **(F)** Quantification of cell apoptosis ratio showed in **(E)**. *n* = 3. Each bar shows as mean ± SD. ^∗^*P* < 0.05; ^∗∗^*P* < 0.01; ^∗∗∗^*P* < 0.001. OD value, optical density value; NS, no significance.
