# Supplementary figures and images for: Honokiol Alleviates Hypertrophic Scar by Targeting Transforming Growth Factor-β/Smad2/3 Signaling Pathway
Source: Front Pharmacol. 2017 Apr 19;8:206. doi: 10.3389/fphar.2017.00206 (PMC5395562; doi:10.3389/fphar.2017.00206)

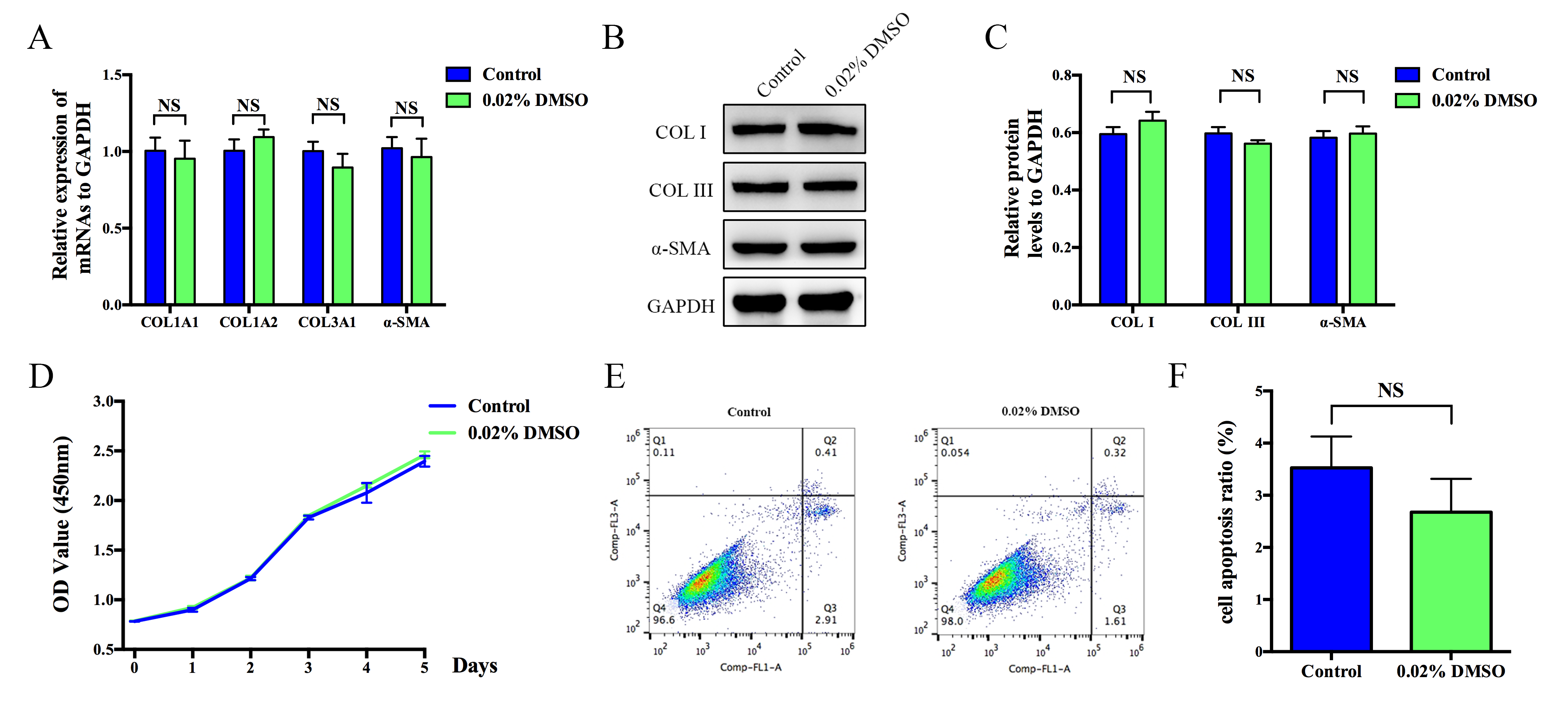

Supplement: FIGURE S1 — Effects of the solvent DMSO at its highest concentration of 0.02% used for HKL preparation. (A) qRT-PCR results of the mRNA levels of COL1A1, COL1A2, COL3A1, and α-SMA after treatment of 0 μg/ml HKL and 0.02% DMSO. GAPDH served as control. n = 3. (B) The effect of 0.02% DMSO on protein levels of COL I, COL III and α-SMA in HSFs by Western blot. GAPDH served as control. (C) Quantification of protein levels in (B) which normalized to the level of GAPDH. n = 3. (D) Assessment of the effect of 0.02% DMSO on HSFs proliferative activity by CCK-8 assay. (E) Assessment of the effect of 0.02% DMSO on cell apoptosis by flow cytometry. (F) Quantification of cell apoptosis ratio showed in (E). n = 3. Each bar shows as mean ± SD. ∗P < 0.05; ∗∗P < 0.01; ∗∗∗P < 0.001. OD value, optical density value; NS, no significance. [file Image_1.TIF]
